# Multicenter study of lumen-apposing metal stents with or without pigtail in endoscopic ultrasound-guided biliary drainage for malignant obstruction—BAMPI TRIAL: an open-label, randomized controlled trial protocol

**DOI:** 10.1186/s13063-022-06106-1

**Published:** 2022-02-25

**Authors:** Albert Garcia-Sumalla, Carme Loras, Vicente Sanchiz, Rafael Pedraza Sanz, Enrique Vazquez-Sequeiros, Jose Ramon Aparicio, Carlos de la Serna-Higuera, Daniel Luna-Rodriguez, Xavier Andujar, María Capilla, Tatiana Barberá, Jose Ramon Foruny-Olcina, Belen Martínez, Miguel Dura, Silvia Salord, Berta Laquente, Cristian Tebe, Sebastia Videla, Manuel Perez-Miranda, Joan B. Gornals, On behalf of Spanish Working Group on Endoscopic Ultrasound Guided Biliary Drainage

**Affiliations:** 1 Endoscopy Unit, Department of Digestive Diseases, Hospital Universitari de Bellvitge, Bellvitge Biomedical Research Institute (IDIBELL), Barcelona, Catalonia Spain; 2 Bellvitge Biomedical Research Institute (IDIBELL), Barcelona, Spain; 3 University of Barcelona, Barcelona, Spain; 4 Endoscopy Unit, Department of Digestive Diseases, Hospital Universitari Mútua Terrassa, Fundació per la Recerca Mútua Terrassa, CIBERehd, Terrassa, Spain; 5 Health Sciences, Universitat Oberta de Catalunya, Barcelona, Spain; 6 Endoscopy Unit, Department of Digestive Diseases, Hospital Clínico Universitario de Valencia, Instituto de Investigación Sanitaria INCLIVA, Valencia, Spain; 7 Endoscopy Unit, Department of Digestive Diseases Endoscopy Unit, Hospital General Universitario de Castellón, Castelló de la Plana, Spain; 8 Endoscopy Unit, Gastroenterology and Hepatology Service, Hospital Ramon y Cajal, IRYCIS, Madrid, Spain; 9 Endoscopy Unit, Department of Digestive Diseases, Hospital General Universitario de Alicante; Instituto de Investigación Sanitaria y Biomédica de Alicante (ISABIAL), Alicante, Spain; 10 Endoscopy Unit, Department of Digestive Diseases, Hospital Universitario Rio Hortega, Valladolid, Spain; 11 Hepato-Bilio-Pancreatic Unit, Digestive Diseases Department, Hospital Universitari de Bellvitge, Bellvitge Biomedical Research Institute (IDIBELL), Barcelona, Catalonia Spain; 12 Clinical Oncology Department, Hospital Duran y Reynalds, Institu Oncologic de Catalunya, Barcelona, Catalonia Spain; 13 Biostatistics Unit, Institute of Biomedical Research of Bellvitge, L’Hospitalet de Llobregat, Barcelona, Spain; 14 Clinical Research and Clinical Trial Unit (UICEC), Clinical Pharmacology Department, Hospital Universitari de Bellvitge, Barcelona, Catalonia Spain

**Keywords:** Biliary drainage, Choledochoduodenostomy, Endoscopic ultrasound, Lumen-apposing metal stent, Malignant biliary obstruction, Plastic stent, Trial

## Abstract

**Background:**

It is unclear whether the insertion of an axis-orienting double-pigtail plastic stent (DPS) through biliary lumen-apposing meal stent (LAMS) in EUS-guided choledochoduodenostomy (CDS) improves the stent patency. The aim of this study is to determine whether this technical variant offers a clinical benefit in EUS-guided biliary drainage (BD) for the management of distal malignant biliary obstruction.

**Methods/design:**

This is a multicenter open-label, randomized controlled trial with two parallel groups. Eighty-four patients with malignant biliary obstruction will undergo EUS-BD (CDS type) using LAMS in 7 tertiary hospitals in Spain and will be randomized to the LAMS and LAMS plus DPS groups. The primary endpoint is the rate of recurrent biliary obstruction, as a stent dysfunction parameter, detected during follow-up. Secondary endpoints: technical and clinical success (reduction in bilirubin > 50% within 14 days of stent placement), safety, and others (number of reinterventions, time to biliary obstruction, prognostic factors, survival rate).

**Discussion:**

The BAMPI trial has been designed to determine whether the addition of a coaxial axis-orienting DPS through LAMS is superior to LAMS alone to prevent stent dysfunction.

**Trial registration:**

ClinicalTrials.govNCT04595058. Registered on October 14, 2020.

**Supplementary Information:**

The online version contains supplementary material available at 10.1186/s13063-022-06106-1.

## Administrative information

**Table:** 

Protocol ID	BAMPI TRIAL (Biliary Apposing Metal Pigtail)
**Short title**	**RANDOMIZED MULTICENTER EUS-BD: LAMS VS LAMS-PIGTAIL**
**Version**	Version 1.3
**Date**	December 2021
**Promoter**	Dr. Joan B. Gornals, MD, PhD Director of Endoscopy Unit Hospital Universitari de Bellvitge – IDIBELL.
**Principal investigator**	Dr. Joan B. Gornals Digestive Endoscopy Unit Hospital Universitari de Bellvitge – IDIBELL c/ Feixa Llarga s/n, L’Hospitalet de Llobregat, BARCELONA, CATALONIA, SPAIN
***Research Fellow***	Dr. Albert Garcia-Sumalla MD - Digestive Endoscopy Unit Hospital Universitari de Bellvitge – IDIBELL, Barcelona
***Steering committee***	Dr. Carme Loras MD, PhD – Hospital Mútua de Terrassa, Barcelona, Spain Dr. Manuel Pérez Miranda MD, PhD – Hospital Universitario Río Hortega, Valladolid, Spain Dr. Joan B Gornals MD, PhD – Hospital Universitari Bellvitge – IDIBELL, Barcelona, Spain
**Trial registration**	**NCT 04595058** ClinicalTrials.gov Registered on October 14, 2020 https://clinicaltrials.gov/ct2/home

## Main text

### Background

Transpapillary stenting by endoscopic retrograde cholangiopancreatography (ERCP) remains the gold standard treatment for malignant biliary obstruction (MBO), but may fail in up to 15% of cases, and it carries the risks of post-ERCP pancreatitis and stent dysfunction secondary to tumor ingrowth and/or overgrowth [1, 2]. For these reasons, since the emergence of Endoscopic Ultrasound-guided biliary drainage (EUS-BD) using electrocautery-enhanced (EE) lumen-apposing metal stents (LAMS), by creating a choledochoduodensotomy (CDS), it has been proposed as a viable alternative to ERCP [3, 4]. Firstly, as a rescue strategy after failed ERCP, in palliative scenarios, or even as a bridge to surgery [5, 6], and then recently, as a first-line modality for MBO in controlled trials [2, 7–9].

Although recent metanalyses and systematic reviews [10–14] have postulated that EUS-BD using LAMS has high technical and clinical success rates, some concerns exist regarding its safety, as non-negligible rates of adverse events (AE) have been reported [15, 16]. Recurrent biliary obstruction (RBO), as a stent dysfunction parameter, is a major issue to consider after EUS-BD using LAMS. Several cases of stent dysfunction (e.g., food or lithiasis impaction, sump syndrome, stent migration) have been documented due to a possible limitation in LAMS design, as the short length of the stent causes the distal flange to become impacted against the opposite biliary wall [10, 17, 18]. The insertion of a coaxial double-pigtail plastic stent (DPS) has been demonstrated to improve LAMS patency and bile flow, by maintaining a vertical orientation in the bile duct. But a recent retrospective study by our group did not encounter sufficient evidence to recommend its routine use [19].

For these reasons, a multicenter randomized controlled trial has been designed to assess whether a coaxial DPS within a biliary LAMS is superior to a single LAMS in EUS-BD (CDS type) for MBO.

### Methods/design

The BAMPI (*B*iliary *A*pposing *M*etal *PI*gtail) trial is a multicenter, open-label, randomized controlled clinical trial with two parallel groups, and with a 1:1 allocation ratio. Eighty patients with MBO will be scheduled for EUS-BD in seven tertiary Spanish hospitals and will be randomized to the LAMS alone or the LAMS plus DPS group. We hypothesize the addition of a prophylactic DPS through LAMS to be associated with fewer episodes of RBO and, consequently, fewer biliary reinterventions (BRI).

Central ethical approval of the study protocol has been confirmed by the Comité Ético de Investigación Clínica (CEIC) del Hospital Universitari de Bellvitge-IDIBELL (ref approval no. ICPS024/20, on November 2020) and we will not begin recruiting at other centers in the trial until local ethical approval has been obtained.

**Fig. 1:**
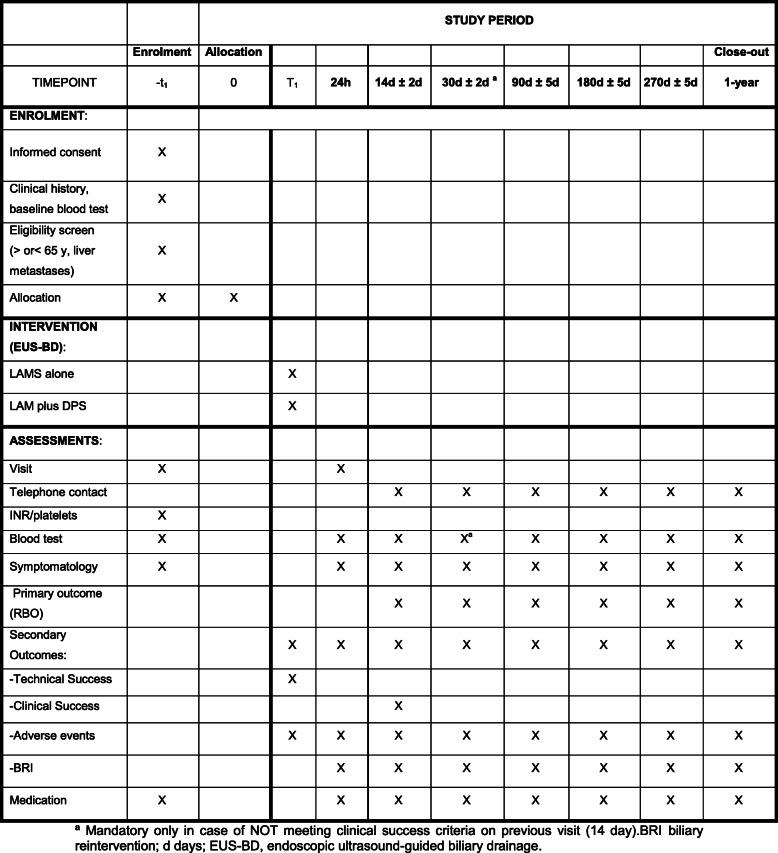
Enrollment, interventions, and assessment in the BAMPI trial (SPIRIT-Figure). ªMandatory only in case of NOT meeting clinical success criteria on a previous visit (14 days).BRI biliary reintervention; d days; EUS-BD, endoscopic ultrasound-guided biliary drainage

A checklist with the recommendations for Interventional Trials (SPIRIT) is attached as Additional file 1: SPIRIT-figure with schedule of enrolment, interventions, and assessments in the BAMPI trial (Fig. 1).

#### Study population: Patient identification and consent

All patients admitted with distal MBO and clinical criteria that justify EUS-BD will be considered for consent. The investigator at each participating center will identify potential patients and will assess the inclusion of the patient in the study, and eligibility either as a first-line BD method, or as a rescue method after failed ERCP. The patient will be correctly informed by personnel knowledgeable about the specifics of the study, who will help to resolve any questions that may arise. The informed consent form will be signed prior to the procedure and a signed copy will be given to the patient. The patient has the right to opt out of the study at any time.

The inclusion and exclusion criteria are listed in Table 1.

**Table 1:** Inclusion and exclusion criteria

**Inclusion criteria:** **Patients eligible for the trial must fulfill all the following at randomization** - Age 18 years or more. - Malignant biliary obstruction with clinical criteria that justifies EUS-guided biliary drainage. - Capable of understanding and signing informed consent form. - Understanding the type of study and complying with the follow-up of complementary tests during the study’s duration.	
**Exclusion criteria:** **Patients with any of the following will be excluded** - Pregnancy or breast-feeding. - Severe coagulation disorder: INR > 1.5 not correctible with administration of plasma and/or platelets < 50,000/mm3. - Maximum cross diameter of the CBD < 10 mm. - Another type of biliary drainage at the time of the procedure (cholecystostomy, percutaneous drainage, etc.). - Failure to sign informed consent form. - Intellectual handicap and unable to understand the nature and possible consequences of the study, unless there is a competent legal representative. - Unable to adhere to subsequent follow-up requirements.	

*CBD* common bile duct, *EUS* endoscopic ultrasound, *INR* international normalized ratio

#### Recruitment

Principal investigators from each center will have the task of presenting strategies to promote enrolment and ensure the target sample size. Prior to the start of the study, a meeting with oncology and digestive surgery teams will be organized with the aim of creating collaboration bridges.

#### Randomization and masking

Patients will be enrolled in this trial by gastroenterologists, surgeons, oncologists, and endoscopists who will evaluate cases in the inpatient wards or outpatient consultation areas. Investigators of each center will be responsible for entering all necessary criteria to an online platform that will generate the randomization sequence, and participants will be randomized with an arbitrary number.

A code list will be generated by our biostatistics department, using R software (v 3.6.3) by randomization with a 1:1 randomization ratio, by blocks, stratified by age (< 65 years old/> 65 years old) and by the presence of liver metastases. The assumption is made that liver metastasis may elevate bilirubin and cholestasis parameters without involving bile duct obstruction. Everyone will be assigned a randomization code along with the treatment that corresponds to it. Once the patient meets the eligibility criteria and has provided informed consent, we will proceed to the allocation of each participant centrally, ensuring allocation concealment, and based on the randomization list. To prevent different subject recruitment rates at the various hospitals from interfering in the development of the study, the entire population will be randomized in blocks of four between the two treatment possibilities.

#### Procedural technique

##### Qualification of centers

This clinical trial will be performed at the endoscopy unit of seven referral centers in Spain. Endoscopists will all be experienced in endoscopic intervention and therapeutics, such as stenting and EUS-guided transmural drainage. To avoid biases derived from the learning curve, only those centers with proven experience using biliary EE-LAMS (EUS-guided CDS) have been invited to participate. To objectify usage data, the database “NRPAL: National Registry of Lumen Apposing Metal Stents Incidences, NCT04059926”, which collects data from all LAMS placed for 1 year, was checked. Minimum requirements were at least 12 LAMS placed in one year for any indication and a minimum experience of 7 EUS-BD using EE-LAMS. All the experienced endoscopists are members of the Spanish Society of Digestive Endoscopy (SEED).

##### General description of the technique

Treatment allocation is to EUS-BD with LAMS alone vs. LAMS plus DPS. All procedures will be performed by experienced endoscopists in EUS-guided transmural stenting. Procedures will be performed under deep sedation or tracheal intubation, in accordance with the directives of each center. Coagulation disorders will be corrected prior to the procedure. In case of INR > 1.5, this will be corrected with the protocol of each institution. In all interventional procedures, CO2 will be used as an insufflation agent. Prophylactic antibiotic therapy will be given in accordance with institutional protocols.

##### EUS-BD with EE-LAMS

Each selected case will ensure a conclusive diagnosis of MBO. If necessary EUS-guided tissue acquisition (TA) will be performed previously to or immediately after the transmural drainage. The TA technique will be at the endoscopist’s choice. Firstly, EUS oversight will be done with the purpose of ruling out features that could jeopardize a transmural BD (such as ascites, long distance common bile duct (CBD)-transducer, or intervening vessels). The extrahepatic bile duct will be identified by using a linear echoendoscope (Fujifilm EG-580-UT, Olympus GF-UCT180), selecting an optimal point for carrying out the EUS-guided intervention. The long scope position will preferably be used to maintain stability and, if necessary, to ease advancing of a guidewire to intrahepatic direction. A small-medium sized LAMS (6, 8, and 10 mm in diameter and 8 or 10 mm in length, Hot-AXIOS™ stent with an electrocautery-enhanced delivery system, Boston Scientific, Marlborough, MA, USA) will be used in all included cases.

The access step will be standardized in two different techniques, as explained in our previous report [19]: (i) freehand style (optional preloaded guidewire), with direct access to the bile duct, and (ii) classic technique, with initial puncture of the extrahepatic bile duct using a 19-gauge needle and advancing a standard guidewire, after which a LAMS is advanced over the guidewire.

In all cases, the biliary LAMS is inserted using the cautery system, and deployed under EUS-guidance without tract dilation. The use of fluoroscopy will be decided upon based on technical considerations and the endoscopist’s opinion.

Selection of LAMS size will be based on bile duct diameter and availability.

##### LAMS plus coaxial DPS procedure

In those patients allocated in the LAMS-DPS cohort, a DPS (preferably 7Fr × 3, 5 or 7 cm, Advanix, Boston Sc) will be placed coaxially through the LAMS, preferably with upward (intra-hepatic) orientation. This selection of small DPS sized should ease the advance and release by offering less friction.

##### Additional interventions

After the LAMS deployment, intra-stent dilation is permitted using balloons at the endoscopist’s discretion.

Other endoscopic procedures such as EUS-guided TA, EUS-guided gastro-enterostomy, stenting (esophageal, duodenal), and EUS-guided celiac plexus blockage/neurolysis performed during the index procedure will be meticulously noted in a specific section in the electronic case report form (eCRF).

##### Gallbladder option

When a CDS is not possible or too risky, a BD rescue by EUS-guided gallbladder transmural drainage may be considered. It is important to note that this should only be offered in cases without an optimal endosonographic window for the creation of CDS (e.g., interposal vessels, CBD < 10 mm, excessive GI tract-CBD distance, scope instability, others). It may be randomized in the same way (LAMS vs. LAMS-DPS group) and followed up according to the protocol. This additional subgroup will be analyzed and documented, but outside of the trial’s main outcomes.

##### Additional comments

No removal of the stents is contemplated due to the malignant condition of the participants. Non-inpatients, they will remain in the center for a minimum of 24 h for clinical observation under clinical supervision.

In case of technical failure for any reason, alternative strategies will be decided upon with the aim of offering the best possible treatment to the patient.

#### Clinical evaluation and follow-up

##### Data collection and calendar

**Fig. 2:**
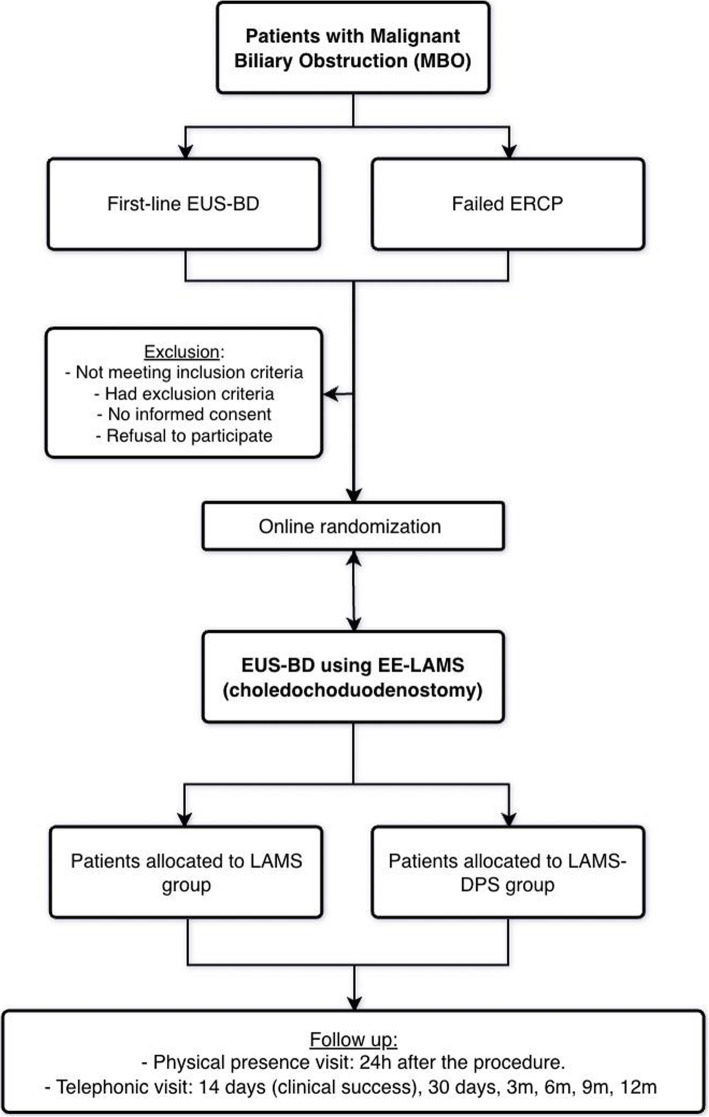
BAMPI trial Flowchart. DPS double-pigtail plastic stent, ERCP endoscopic retrograde cholangio-pancreatography, EUS-BD endoscopic ultrasound-guided biliary drainage, EE-LAMS, electrocautery-enhanced, lumen-apposing metal stent

The collecting of clinical information of the patients will begin at the outset (baseline) and will continue with follow-up as established and defined in the study. Data will be collected at baseline visit, indexing procedure 24 hours later, and at days 14, 30, 90, 180, 270, and 1-year post-randomization. Collected data include primary, secondary, and additional endpoint data, demographics, comorbidities, oncological data, laboratory test findings, technical details, and clinical data during follow-up (Figs. 1 and 2).

AEs will be noted from the beginning of the test until the conclusion of follow-up by means of scheduled controls and will be handled and treated in accordance with the directives of the patient’s medical team. All additional tests and interventions will be duly documented. Any instances of death during the follow-up will be investigated to rule out possible relation to the endoscopic procedure. Such occurrences will also be recorded in the CRF.

If there is clinical suspicion of obstruction or migration of the stent, an upper endoscopy will be carried out. Based on the findings of this procedure, the problem will be resolved in accordance with the directives of the intervening endoscopist. Any additional procedure or endoscopic intervention will be duly documented.

All data will be collected by research personnel knowledgeable in the use of the eCRF (data capturing software, redCAP). Collected patient data will be de-identified, meaning that personal information will not be stored in the main database. All data will be stored for 7 years after publication or as requested in standard operating procedure from individual sites. Only data relevant to the study as outlined in this protocol will be collected by the research team.

Due to the social and health crisis arising from the SARS-CoV-2 pandemic and taking into account the likely frailty of the candidates to be enrolled in the study, the protocol contemplates restrictions on physical presence visits. Only the indexing procedure and the 24-h post-procedure visits will require an on-site visit since a minimum one-day hospital admission is mandatory.

A BAMPI trial flowchart is provided (Fig. 2).

#### Definitions

Technical success is defined as successful placement of the stents (LAMS, DPS, or both) between the extrahepatic bile duct and gastrointestinal lumen by endoscopy, endosonography, and, optionally, fluoroscopy.

Clinical success is defined according to the TOKYO criteria as a 50% decrease in or normalization of total serum bilirubin level within 14 days of index procedure (stent placement). For cholangitis without obstructive jaundice (e.g., segmental cholangitis, cholecystitis), success can be defined as cessation of antibiotics or a 50% decrease in or normalization of levels of blood inflammatory markers within 14 days of stent insertion [20].

RBO is defined as a composite endpoint of stent dysfunction (either occlusion or migration). See Table 2 (outcomes) for complete information [20].

**Table 2:** Primary and secondary endpoints

**Primary endpoint:** - Rate of recurrent biliary obstruction (RBO) between the two strategies (LAMS with and without coaxial DPS), detected during follow-up. *RBO is associated with a stent dysfunction (an endpoint of either occlusion^a^ or migration^b^). Tokyo criteria - Clinical recurrence (jaundice, fever, suspicious colangitis, pruritus). - Recurrence of cholestasis parameters (Any increase in GGT/ALP or bilirubin from its lowest level post-index procedure). Both WITH evidence of biliary obstruction on imaging (dilation on US/CT/MRI) or endoscopic findings suggesting it ^c^.	
**Secondary endpoints:** - Technical success defined as successful stents (LAMS, DPS, or either) between the extrahepatic biliary duct and the upper gastrointestinal tract determined by endoscopy, endosonography, or fluoroscopy. - Clinical success defined as > 50% decrease in bilirubin at 14 days from stent placement. For cholangitis, clinical success is defined as cessation of antibiotics or normalization of levels of blood inflammatory markers within 14 days of stent placement. - Safety, as defined per the ASGE lexicon/Tokyo criteria for endoscopic AEs and divided into early adverse events (within 14 days of index procedure) and delayed AE (> 14 days). - Additional outcomes: t-RBO, BRI, procedure time, and survival/mortality rates.	

*AE* adverse event, *ALP* alkaline phosphatase, *ASGE* American Society of Gastrointestinal Endoscopy, *BRI* biliary reintervention, *CT* computed tomography, *ERCP* endoscopic retrograde cholangio-pancreatography, *EUS* endoscopic ultrasound, *GGT* gamma-glutamyl transferase, *RBO* recurrent biliary obstruction, *US* ultrasound, *MRI* magnetic resonance imaging

^a^Stent occlusion defined as elevation of inflammatory parameters/cholestasis evidence along with biliary dilation on imaging studies or endoscopic findings. Tokyo criteria [20]

^b^Stent migration defined as presence of completely/partially migrated stent at the time of endoscopic reintervention with evident stent dysfunction

^c^Causes of stent occlusions: tumor ingrowth/overgrowth, biliary sludge, food impaction, hemobilia, kinking of CBD to stent). Tokyo criteria [20]

Time to RBO (t-BO) or stent patency is defined as the time from stent placement until the point when symptoms associated with occlusion or migration are observed.

BRI is defined as the need to perform a new therapeutic maneuver on the bile duct due to RBO. A distinction must be made between:

- Endoscopic biliary reintervention (e-BRI): endoscopic procedure with the aim of optimizing the transmural BD. It includes stent cleaning, stent change, additional stent insertion, or any other stent-related endoscopic maneuver. For specific information, see Rescue options below.
- Radiological biliary reintervention (r-BRI): interventional radiological procedure with percutaneous access, with the aim of repermeabilizing the obstructed endoscopic BD.

#### Safety reporting (AEs)

AE is any unwanted medical event, injury, or clinical (e.g., signs, symptoms, or abnormal laboratory results) suffered by patients during the study, whether related to the endoscopic procedure or stent. All AEs (reported by medical staff or patients) must be documented.

All serious adverse events (SAEs) must be notified to the principal investigator within 3 days. In the event of a death, this notification will be made within less than 24 h.

AEs will be rated as mild (hospitalization for 1-3 days), moderate (hospitalization for 4–10 days, ICU admission for 1 night, endoscopic/radiological intervention), severe (hospitalization > 10 days, ICU admission > 1 night, or necessity for surgery), or fatal, in accordance with the nomenclature for AEs in endoscopy (American Society for Gastrointestinal Endoscopy (ASGE) Workshops 2010) [21].

AEs associated with endoscopic biliary stents (e.g., pancreatitis, non-occlusion cholangitis, cholecystitis, bleeding, perforation, bile leakage) are defined by the standardized reporting system for endoscopic biliary stent placement (Tokyo criteria, 2018) [20, 21]. It is important to note that RBO is not classified as an AE, but as a consequence of stent occlusion or migration.

Non-occlusion cholangitis will be defined as fever which continues longer than 24 h without dilation of the drained duct. It may require medication, hospitalization, or intervention.

The determination as to what is an AE related to the procedure or the medical device (procedure-related and stent-related) and what is not will be made by the medical team and local investigator, with the final approval of the principal investigator (JG).

The AE will be chronologically differentiated as pre-procedure, intra-procedure, post-procedure, or early AE (up to 14 days from the index procedure), and, finally, late AE (after 14 days) according to the ASGE lexicon guideline [21]. Additionally, another phone call has been added at 30 days, to ensure that all stent-related AEs will be detected, as suggested by Tokyo criteria [20].

To clarify the causal relationship and grading of AEs, MEDDEV guidelines will be consulted. Additional file 2 includes all AE definitions based on the MEDDEV 2.12 guidelines (rev 8, July 2019) “Guidelines on medical devices: Clinical investigations: Serious Adverse event reporting under Directives 93/42/EEC and 90/385/EEC”, regarding safety in clinical research with medical devices, which are determined according to the causal relationship and/or severity of AEs.

#### Outcomes

The primary outcome is the rate of RBO after the index procedure, detected during follow-up. As noted above (in the “Definition” section), this is a direct indicator of stent dysfunction, and it is defined as a composite point of either biliary occlusion or stent migration.

The secondary outcomes include technical and clinical success (> 50% decrease in bilirubin at 14 days from stent placement), safety data, and other patient-relevant outcomes (time to RBO, number of reinterventions, procedural time, and mortality rates).

Completed outcomes definitions are presented in Table 2.

#### Sample size calculation

The sample size calculation is based on the primary hypothesis of detecting significant differences in the rates of RBO and BRI between the LAMS and LAMS plus DPS groups (assessing for superiority).

- Null hypothesis (H0): πLAMS = πLAMS-DPS
- Alternative hypothesis (H1): πLAMS ≠ πLAMS-DPS

Based on recently published data, we estimate a recurrent biliary obstruction rate of 20% vs. 1% for the LAMS alone vs. LAMS-DPS groups, respectively up during the follow-up period [13, 19, 22]. To achieve a statistical power of 80% with a two-sided type I error of 5%, a total of 80 patients (40 in each arm) is required to reject the null hypothesis. https://www.stat.ubc.ca/~rollin/stats/ssize/b2.html

Considering a 5% dropout rate, a final sample of 84 patients (42 patients in each arm) is needed.

#### Statistical analysis

Statistical analysis will be carried out with the R statistical software (version 3.6.2) supervised by the Biostatistics team of our research institute (IDIBELL).

Intention-to-treat and per-protocol analysis will be carried out. All study variables will be presented for stent groups and in total, using descriptive statistics in accordance with the nature of the variable. Thus, continuous variables will be described indicating the number of non-missing observations, mean, standard deviation, minimum, first quartile, median, third quartile, and maximum. The categorical variables will be described indicating the number of non-missing observations and the percentages of the different categories.

Realization of the aim of the study will be analyzed by comparing the cumulative incidence of RBO (as a stent dysfunction parameter) in the LAMS cohort versus LAMS-DPS cohort using a Chi-Square test. The magnitude of the effect will be estimated through an incidence ratio and relative risk with 95% confidence interval. The main analysis will be replicated in an adjusted way using a binomial regression model. Age, sex, oncological status, and comorbidities will be taken as adjustment variables.

Safety will also be evaluated by describing the number and type of AE in each study group, and the incidence will be compared using a chi-square test. In addition, the survival of patients will be analyzed according to the two proposed strategies until the end of follow-up, describing the Kaplan-Meyer curves and comparing them using the LogRank test. The level of statistical significance is established at *p* value < 0.05.

##### Subgroup analysis

The main analysis will also be carried out in the following subgroups:

- Age < 65 vs. age > 65.
- Presence of liver metastasis vs. Absence of liver metastasis.

#### Data management

Throughout the study, the promoters will monitor the quality of the trial with special attention to protocol deviations and the quality of the data entered in the database. Scheduled video calls will be performed by the primary investigator (JG) and/or research fellow (AGS). These audits will occur at 6 months post-enrollment and at the end of the study period. AGS will send a monthly newsletter communicating the trial progress to all investigator sites. At the end of the trial, a meeting will be held to consider the data management report. This report will describe the deviations from the protocol identified in each of the patients. These deviations will be classified as major or minor, and those patients with major deviations will be excluded from the protocol analysis. After the meeting, the suitability of the database for analysis will be considered, and the database will then be closed.

#### Statistical analysis plan

The statistical analysis plan will be finalized before the close of the database. This plan will include all the analyses described and others, mainly on the sensitivity of the results and the management of the missing data. If there is some deviation in the plan of statistical analysis regarding the main variable, an addendum to the protocol will be made. No changes will be made to the original analysis plan once the database is closed.

No interim analysis is planned given the short accrual time, relatively small sample size, and short follow-up period.

#### Other considerations

##### Rescue by e-BRI and cross-over

Depending on the initial endoscopic treatment carried out, in case of stent dysfunction or RBO, cross-over rescue treatment may be considered when the initial per-protocol strategy fails:

- In LAMS cohort: adding a co-axial DPS through LAMS.
- In LAMS-DPS cohort: removing DPS and leaving LAMS or adding another coaxial stent, either fully-covered self-expandable metal stent or another DPS.

These potential cross-over options are considered in the e-BRI, described in a previous section (see definitions). In all these situations, the follow-up will be maintained until the end of the study in accordance with the protocol. Alternatively, if an endoscopic technique is not possible, other options (such as radiological intervention) will be offered.

##### Withdrawal

Withdrawal from the study will result from any AE or other clinical condition of the patient which, at the clinician’s discretion, warrants such, or from pregnancy, or at the expressed wishes of the patient. Withdrawal from treatment will not mean suspension of the study, given that follow-up will be maintained until the end of the study in accordance with the protocol.

#### Ethical aspects and confidentiality

The protocol will be approved by the CEIC of each participating hospital as well as that of the coordinating center (Hospital Universitari de Bellvitge). The study researchers will carry out their tasks in compliance with ethical principles of clinical research established in the Declaration of Helsinki, and with the norms of Good Clinical Practices.

The study is considered a clinical trial with a low intervention level since it compares two standard treatments with each other. Furthermore, all the medical devices used have CE marking with authorization for their use in endoscopic biliary drainage. For these reasons, the contracting of a specific civil liability policy is not required in accordance with the provisions of current legal regulations, complying with the assumptions established for coverage by the assistance policy.

Before the inclusion of the patient in the trial, written informed consent will be requested. In relation to the study data, we will follow the provisions of Organic Law 3/2018 of December 5th on “Protection of Personal Data”.

Steering committee will meet before, at the half, and after of the recruitment starting and will provide supervision on the progress of the trial, adherence to the protocol, patient safety, and consideration of new information relevant to the research hypothesis. Any amendment or change made to the original protocol must be accepted by the steering committee.

Comité Ético de Investigación Clínica del Hospital Universitari de Bellvitge --IDIBELL (Barcelona; ref approval no. ICPS024/20) has reviewed and accepted this protocol before the recruitment starting. Data collected during the research will be kept strictly confidential and only accessed by investigators. An individual trial identification number and participant details will be stored on a secure database. Any subsequent amendments of the protocol need to be approved by the relevant ethical bodies before implementation. At the end of the study, a report will be sent to the Ethic committee.

#### Data Monitoring Committee

Data Monitoring Committee (DMC) will be created ad hoc. The composition of DMC will be: a biostatistician, a pharmacovigilance specialist, and an external expert on the subject, whom are not involved in the execution of the clinical trial. The meeting will take place when the first half of included patients complete the study. The aim of this DMS is: to review the adverse events and the main variable to assess the risk-benefit and futility. The DMC may decide to hold a second meeting to further evaluate the benefit-risk balance. The inclusion of the patients in the clinical trial will not be stopped during the DMC procedure.

#### Publication of results

There is a commitment to publish the results of this study in high-impact international journals, should the results be of sufficient scientific interest. No patient names will appear in any article, and no one, with the exception of the researchers in this study and the members of the hospital ethical committees, will have access to the data, in accordance with the Law on the Protection of Data of a Personal Nature.

### Discussion

Currently, in vogue in the recent literature is reporting that EUS-BD (CDS type) using EE-LAMS is an effective and safe technique for biliary decompression in patients who failed ERCP [5, 6, 10–12, 23]. Some prospective studies or protocol trials have even been designed to assess CDS using LAMS as the primary treatment modality for distal MBO over ERCP [2, 7–9].

In fact, if it is shown that EUS-CDS is superior to ERCP, in terms of stent patency and safety, for the first-line drainage of MBO, it is expected that the first-line BD method will be changed from ERCP to EUS-CDS, and that interruption of chemotherapy due to stent dysfunction could be avoided [9]. Additionally, even though percutaneous transhepatic BD (PTBD) may be considered as another alternative option after failed ERCP, some recent reports have claimed that EUS-BD with LAMS is superior to PTBD in terms of clinical success, safety, cost, and overall survival [24].

The introduction of these specific biliary LAMS represents a great technical improvement in EUS-BD of distal MBO [3, 4]. The cautery-assisted stent eases the transmural technique, avoiding complexity, and failure risk linked to the devices exchanging or ostomy dilation, even without fluoroscopy guidance.

Even so, in two recent papers, including a meta-analysis, showing that LAMS and SEMS are comparable in terms of efficacy, safety, and reintervention rates for EUS-CDS, the authors concluded that further investigation is required, above all in adverse events [13, 25].

Stent dysfunction of biliary LAMS in EUS-CDS is one of the main concerns, and it has been reported in up to 26.3% of cases [15, 17, 18]. Unlike in pancreatic fluid collection [26], the prophylactic placement of a DPS within a biliary LAMS could offer two hypothetical advantages to prevent biliary AEs as RBO: (i) as an axis-orienting stent to maintain a non-perpendicular axis of the LAMS in the CBD; (ii) decreasing the risk of migration, limiting the possibility of LAMS slipping out. In a US multicenter retrospective study, El Chafic et al. proposed this technical variant, because RBO developed in significantly more patients with LAMS alone compared to LAMS plus DPS (50% vs. 11.8%; p=0.02) [23]. Similarly, two recent papers, including a large cohort from the UK and Ireland, revealed lower rates of cholangitis and BRI (0% vs 12.2%, *p* .03) in the DPS-LAMS group [22, 27]. None of these previous studies were specifically designed for comparing the usefulness of the coaxial stent.

Recently, our group published a retrospective comparative study assessing the hypothetical benefit of a DPS within a biliary EE-LAMS in EUS-CDS. The DPS plus LAMS group had a higher clinical success rate, non-significantly lower AE rate, and longer significant procedural time. Curiously, the RBO rate was a little bit higher in the DPS-LAMS group [19]. Therefore, these results did not support this technical variant being used routinely, and it was a time-consuming approach.

Why the need to carry out a larger prospective and randomized study?

In case of confirmation that inserting a DPS within a LAMS during a EUS-CDS reduces the need for re-interventions, improves the biliary stent patency, and prevents delays in the oncological management, this would surely have an impact on the oncological outcomes and survival rates. To date, there is no prospective data about this issue.

The BAMPI trial will include seven referral centers, experts in EUS-guided transmural drainage, with proven experience in EUS-BD using EE-LAMS. Hospital Universitari de Bellvitge has a leadership role in centralizing decisions in case of controversies, and in limiting heterogeneity.

In conclusion, this is the first multicenter randomized controlled trial designed to clarify whether the DPS plus LAMS strategy should be recommended routinely during a EUS-CDS in distal MBO. The results of this trial will have an impact on the clinical practice in treating patients with biliopancreatic malignancies.

## Trial status

Protocol of submitted version, number, and date: Number 1.3; date December 2021.

Recruitment: Start date November 17, 2020, and recruitment will be completed by October 2022.

Revision chronology:

a- BAMPI, August 2020, original: version 1.0, first draft of the study protocol.
b- BAMPI, November 2020, amendment n° 1: version 1.1.

- Main amendments: (i) to clarify: The study is considered a clinical trial with a low intervention level. To contract a specific civil liability policy is not required in accordance with the provisions of current legal regulations.

c- BAMPI November 2021, amendment n°2: version 1.2.

- Minor changes: addition of a new rescue variant technique. If CDS is not possible (due to interposing vessels, significant distance), gallbladder drainage will be considered.

d- BAMPI December 2021, amendment n°3: version 1.3 – definitive.

- Minor changes: extension of the recruitment period (until October 2022).

## Supplementary Information

**Additional file 1.** SPIRIT checklist.

**Additional file 2.** BAMBI Safety definitions

## Data Availability

Not applicable.

## Abbreviations

AE: Adverse event
ASA: American Society of Anesthesiologists’ classification
BD: Biliary drainage
BRI: Biliary reintervention
CBD: Common bile duct
CEIC: Clinical research ethics committees
CRF: Case report form
DPS: Double-pigtail plastic stent
EE: Electrocautery-enhanced
EUS: Endoscopic ultrasound
EUS-BD: Endoscopic ultrasound-guided biliary drainage
IDIBELL: Bellvitge Biomedical Research Institute
LAMS: Lumen-apposing metal stent
MRI: Magnetic resonance imaging
MBO: Malignant biliary obstruction
PTBD: Percutaneous biliary drainage
RBO: Recurrent biliary obstruction
SEED: Spanish Society of Digestive Endoscopy
TA: Tissue acquisition
