# Correction to: Multicenter study of lumen-apposing metal stents with or without pigtail in endoscopic ultrasound-guided biliary drainage for malignant obstruction—BAMPI TRIAL: an open-label, randomized controlled trial protocol

**DOI:** 10.1186/s13063-022-06138-7

**Published:** 2022-03-14

**Authors:** Albert Garcia-Sumalla, Carme Loras, Vicente Sanchiz, Rafael Pedraza Sanz, Enrique Vazquez-Sequeiros, Jose Ramon Aparicio, Carlos de la Serna-Higuera, Daniel Luna-Rodriguez, Xavier Andujar, María Capilla, Tatiana Barberá, Jose Ramon Foruny-Olcina, Belen Martínez, Miguel Dura, Silvia Salord, Berta Laquente, Cristian Tebe, Sebastia Videla, Manuel Perez-Miranda, Joan B. Gornals, On behalf of Spanish Working Group on Endoscopic Ultrasound Guided Biliary Drainage

**Affiliations:** 1 Endoscopy Unit, Department of Digestive Diseases, Hospital Universitari de Bellvitge, Bellvitge Biomedical Research Institute (IDIBELL), Barcelona, Catalonia Spain; 2 Bellvitge Biomedical Research Institute (IDIBELL), Barcelona, Spain; 3 University of Barcelona, Barcelona, Spain; 4 Endoscopy Unit, Department of Digestive Diseases, Hospital Universitari Mútua Terrassa, Fundació per la Recerca Mútua Terrassa, CIBERehd, Terrassa, Spain; 5 Health Sciences, Universitat Oberta de Catalunya, Barcelona, Spain; 6 Endoscopy Unit, Department of Digestive Diseases, Hospital Clínico Universitario de Valencia, Instituto de Investigación Sanitaria INCLIVA, Valencia, Spain; 7 Endoscopy Unit, Department of Digestive Diseases Endoscopy Unit, Hospital General Universitario de Castellón, Castelló de la Plana, Spain; 8 Endoscopy Unit, Gastroenterology and Hepatology Service, Hospital Ramon y Cajal, IRYCIS, Madrid, Spain; 9 Endoscopy Unit, Department of Digestive Diseases, Hospital General Universitario de Alicante; Instituto de Investigación Sanitaria y Biomédica de Alicante (ISABIAL), Alicante, Spain; 10 Endoscopy Unit, Department of Digestive Diseases, Hospital Universitario Rio Hortega, Valladolid, Spain; 11 Hepato-Bilio-Pancreatic Unit, Digestive Diseases Department, Hospital Universitari de Bellvitge, Bellvitge Biomedical Research Institute (IDIBELL), Barcelona, Catalonia Spain; 12 Clinical Oncology Department, Hospital Duran y Reynalds, Institu Oncologic de Catalunya, Barcelona, Catalonia Spain; 13 Biostatistics Unit, Institute of Biomedical Research of Bellvitge, L’Hospitalet de Llobregat, Barcelona, Spain; 14 Clinical Research and Clinical Trial Unit (UICEC), Clinical Pharmacology Department, Hospital Universitari de Bellvitge, Barcelona, Catalonia Spain

**Correction to: Trials 23, 181 (2022)**

**https://doi.org/10.1186/s13063-022-06106-1**

Following the publication of the original article [1], we were notified that the below statement needed to be added to the Acknowledgements section:

‘We thank CERCA Programme / Generalitat de Catalunya for institutional support’.

The original article has been corrected.
